# Itraconazole Inhibits the Growth of Cutaneous Squamous Cell Carcinoma by Targeting HMGCS1/ACSL4 Axis

**DOI:** 10.3389/fphar.2022.828983

**Published:** 2022-02-15

**Authors:** Congcong Xu, Yating Zhuo, Yunyao Liu, Hao Chen

**Affiliations:** ^1^ Institute of Dermatology, Chinese Academy of Medical Sciences and Peking Union Medical College, Nanjing, China; ^2^ State Key Laboratory of Natural Medicines, School of Basic Medicine and Clinical Pharmacy, China Pharmaceutical University, Nanjing, China

**Keywords:** itraconazole, HMGCS1, ACSL4, ferroptosis, cutaneous squamous carcinoma

## Abstract

**Background:** Cutaneous squamous cell carcinoma (cSCC) is a common cutaneous cancer with increasing incidence. Itraconazole has been identified as a potential anticancer drug candidate. However, the role of itraconazole in cSCC was still unclear. Our objective is exploring the therapeutic potential of itraconazole in cSCC and investigate its molecular mechanism.

**Methods:** The anti-proliferation effect of itraconazole was tested with CCK-8 assay and clone formation assay. Cell cycle distribution and apoptosis rate were detected using flow cytometry and TUNEL assay, respectively. Transcriptomic and proteomic analyses were used to explore the underlying anti-cancer mechanism. Luciferase reporter assay was used for promoter activity. Reactive oxygen species (ROS), lipid peroxidation and iron accumulation were examined. The *in vivo* efficacy of itraconazole was assessed in a xenograft model.

**Results:** Itraconazole inhibited the cell proliferation, induced apoptosis and blocked cell cycle of cSCC cells. An integrated analysis of transcriptomic and proteomic analyses identified that 3-hydroxy-3-methylglutaryl-CoA synthase 1 (HMGCS1) and acyl-CoA synthetase long-chain family member 4 (ACSL4) were significantly upregulated in A431 cells treated with itraconazole. HMGCS1 silencing reversed the antiproliferative activity of itraconazole in A431 cells. Dual-luciferase assay showed that itraconazole could promote HMGCS1 transcription. HMGCS1 silencing abated the expression of ACSL4 in A431 cells. The level of ROS, lipid peroxidation, as well as iron accumulation were increased by itraconazole. Moreover, treatment with itraconazole impeded tumor growth in A431-bearing mice.

**Conclusion:** We proved itraconazole inhibits the growth of cSCC by regulating HMGCS1/ACSL4 axis.

## 1 Introduction

Cutaneous squamous cell carcinoma (cSCC) is the second most common skin malignant tumor (Konicke et al., 2018). With its incidence rising over the past years, cSCC constitutes 15%–20% of cutaneous malignancies (Ding et al., 2018; Que et al., 2018). Surgical excision, the main treatment option, is efficiency only at early-stage and clearly defined cSCC (Hughley and Schmalbach, 2018). Other treatment modalities including cryotherapy and imiquimod are related to poor outcome and high rate of recurrence (Keyal et al., 2019). The prognosis of high-grade and metastatic cSCC is still poor (Que et al., 2018). Therefore, it is urgently needed to search more effective therapeutic strategies for cSCC.

Itraconazole is an FDA-approved broad-spectrum antifungal drug that has been used clinically for treating fungal diseases over 3 decades. Researchers have discovered that itraconazole possesses a novel antitumor effect. It has superior anticancer activity against basal cell carcinoma (BCC), non-small lung cancer, prostate cancer, breast cancer, esophageal cancer, medulloblastoma, glioblastoma, etc. (Aftab et al., 2011; Antonarakis et al., 2013; Liu et al., 2014; Pierard-Franchimont et al., 2015; Wang et al., 2017). The results of multiple clinical trials in various cancers confirmed the possibility of itraconazole as a chemotherapeutic agent (Ban et al., 2020). Itraconazole reduced BCC tumor volume via the hedgehog (Hh) signaling pathway in a phase II trial (Kim et al., 2014). Sequential treatment with arsenic trioxide and itraconazole was a workable option for metastatic BCC (Ally et al., 2016). A phase II study with high dose itraconazole demonstrated its exhibited anticancer capability in metastatic castration-resistant prostate cancer (Antonarakis et al., 2013). Treatment with itraconazole plus pemetrexed achieved longer survival compared to pemetrexed alone in progressive non-small cell lung cancer patients (Rudin et al., 2013). The antitumor properties of itraconazole are mediated by a variety of mechanisms. Oral itraconazole, as an Hh pathway inhibitor, reduced tumor size of BCC in Ptch1 ± mice (Kim et al., 2010). Itraconazole induced autophagic progression of glioblastoma cells *in vitro* and *in vivo* (Liu et al., 2014). Itraconazole might have a beneficial effect on colon cancer patients by inhibiting transketolase and inducing autophagy (Shen et al., 2021). The metabolism pathway of specialized pro-resolving mediators was the antitumor mechanism of itraconazole in cervical cancer cells (Isono et al., 2021). Itraconazole had potent therapeutic effect on esophageal cancer, partially via HER2/AKT signaling (Wei Zhang et al., 2021). In a xenograft model of non-small cell lung cancer, itraconazole exerted antiangiogenic efficacy by inhibiting endothelial cell proliferation (Aftab et al., 2011). The anti-hepatocellular carcinoma effect of itraconazole was potential through ROS, Wnt/catenin, Hh, AKT/mTOR/S6K (Wenping Wang et al., 2020). Itraconazole suppressed pancreatic cancer through TGF-β/SMAD2/3 signaling (Ke Chen et al., 2018). Ying Xu et al. found that itraconazole could attenuate the stemness of nasopharyngeal carcinoma cells via sequestering iron in lysosome and then triggering ferroptosis (Xu et al., 2021). It still remains unclear whether itraconazole is effective for cSCC.

Ferroptosis is a recently identified mode of cell death which is driven by iron-dependent lipid peroxidation (Jiang et al., 2021). The increased malondialdehyde (MDA) and iron levels, up-regulated cyclooxygenase-2 (COX-2), and depleted glutathione (GSH) levels are considered as the major biomarkers of ferroptosis (Stockwell et al., 2017). Ferroptosis has been recognized as a key mechanism for cell death associated with metabolism, redox status, and multiple diseases (Stockwell et al., 2017). Excessive ferroptosis has been implicated in ischemia-reperfusion injury and neurodegeneration, whereas abnormal ferroptosis leads to cancer (Lei et al., 2020). Ferroptosis has also been observed in several cancers including head neck squamous cell carcinomas (Ying Wang et al., 2020). Ferroptosis-based therapies, including small molecules and gene technology, have shown anticancer application potential (Ying Wang et al., 2020). Artesunate was used to kill head and neck cancer cells *via* activating ferroptosis (Eling et al., 2015). Gene-knockdown or gene-transfection of ACSL4 and others ferroptosis-related genes have been proved to be promising ways for targeted therapies (Ying Wang et al., 2020; Jie Zhang et al., 2021).

In this study, we established the tumor suppressive role of itraconazole in cSCC, and combined the use of an RNA sequencing (RNA-Seq) database and Label-free proteomic quantification to investigate the underlying mechanism. The findings provided an experimental basis for the use of itraconazole in the treatment of cSCC.

## 2 Materials and Methods

### 2.1 Cell Culture and Reagents

Human cSCC cell A431 and Colo16 were used in our study. For details, please see the Supplementary Material.

### 2.2 Cell Viability Assay

A431 and Colo16 cells were plated in 96-well plates (4 × 10^3^ cells/well), and were incubated overnight and then treated with indicated itraconazole. After indicated time (36, 48, 72 h), 10 μl of CCK-8 solution was added to each well. Absorbance was measured at 450 nm.

### 2.3 Colony Formation Assay

A431 and Colo16 cells were seed at 2,000 cells/well in 6-wells plates and incubated overnight to make them adherent. After that, cells were treated with itraconazole for 48 h and then incubated for 2 weeks. Cells were washed twice with PBS, fixed in methanol, stained with crystal violet, washed and air dried. Cell clusters with more than 50 cells were counted as clones under an inverted microscope.

### 2.4 Cell Cycle

A431 and Colo16 cells were harvested and then fixed in 70% ethanol at 4°C overnight. Cells were washed with cold PBS, resuspended in PBS (500 µl) containing PI (25 µl) and RNase A (10 µl) and incubated for 30 min at 37°C in dark. DNA levels were measured on a NovoCyte flow cytometer, and analyzed using NovoExpress.

### 2.5 Annexin V/PI Staining Assay

Harvested A431 and Colo16 cells were washed twice with PBS, and stained with Annexin V/PI Cell Apoptosis Detection Kit (Vazyme Biotec, Nanjing, China, A211-02), according to the instructions. Analysis was performed by a NovoCyte flow cytometer and NovoExpress software.

### 2.6 Tunel Assay

The TUNEL assay was carried out using TUNEL FITC Apoptosis Detection Kit (1:200; Vazyme, Nanjing, China, A111-01). The details are described in the Supplementary Material.

### 2.7 RNA Isolation and Transcriptome Analyses

A total of three pairs of A431 control cells and A431 cells with itraconazole (1 μM, 48 h) were used for microarray assay to find differentially expressed mRNAs. The detailed procedure is described in the Supplementary Material.

### 2.8 Protein Extraction and Quantification

A total of three pairs of A431 control cells and A431 cells with itraconazole (1 μM, 48 h) were used for the Label-free quantification assay to determine differentially expressed proteins. The detailed procedure is described in the Supplementary Material.

### 2.9 Real-Time PCR Analysis

The details of Real-time PCR analysis are described in the supplementary materials and methods.

### 2.10 Western Blotting Analysis

The details of this part are described in the Supplementary Material.

### 2.11 Plasmid Transfection

Lentiviral systems were used to knockdown human HMGCS1 expression in A431 cells, with HEK293T cells used for viral packaging. HEK293T cells were transfected with pLKO.1 vector, pLKO.1 shHMGCS1 and packaging mix (pMD2G and psPAX2) using Lipofectamine™ 2000 Transfection Reagent (Invitrogen, Carlsbad, CA, United States, 11668019) as described as previously (Qiang et al., 2017).

### 2.12 Dual-Luciferase Reporter Activity Assay

Cells were transfected with pGL3 HMGCS1-Luc, pGL3 HMGCS1-Luc 200 bp mutation, pGL3 HMGCS1-Luc 500 bp mutation, pGL3 HMGCS1-Luc 1,000 bp mutation and pRL-TK (Promega, Madison, WI, United States) using Lipofectamine™ 2000 Transfection Reagent based on the manufacturer’s instructions. Itraconazole or DMSO was added 6 h after transfection. Cells were harvested at 48 h after transfection. Luciferase reporter assays were performed as previously described (Qiang et al., 2016).

### 2.13 Reactive Oxygen Species

The DCFH-DA was diluted in serum-free DMEM medium to a final concentration of 10 μM. Harvested A431 and Colo16 cells were suspended in diluted DCFH-DA, and incubated in a cell incubator at 37°C for 20 min with mixed upside down every 5 min. Cells were washed with serum-free DMEM twice to remove excess DCFH-DA. The level of ROS was detected by NovoCyte flow cytometer (vide supra).

### 2.14 Detection of Cholesterol Concentration, Malondialdehyde Level and Iron Accumulation

The generation of MDA in A431 cells with or without indicated itraconazole was examined by MDA Assay Kit (Applygen, E2019). The levels of iron in A431 cells with or without indicated itraconazole were determined using Iron Colorimetric Assay Kit (Applygen, E1042). The levels of MDA and iron were corrected by intracellular protein concentration. The result in each group was normalized to the control group.

### 2.15 Human Cutaneous Squamous Cell Carcinoma Xenograft Mice Model Study

Female BALB/c nude mice (6 weeks old and 18–22 g weight) were purchased from the Model Animal Research Center of Nanjing University. A431 cells (5 × 10^6^) in cold DMEM (50 μl) were mixed with Matrigel (50 μl) and injected into mice subcutaneously. After 6 days, the tumor volume was measured and the mice were assigned to three groups. Mice were treated with either normal saline or itraconazole (40 mg/kg oral twice daily; 80 mg/kg oral twice daily). Tumor were measured with a caliper every other day, and tumor volume was calculated as follows: tumor volume (mm^3^) = D/2 × d^2^, where D is the longest diameter while d is the shortest diameter. After 24 days after transplantation, the mice were sacrificed, and the tumor and liver tissues were isolated and the tumor weight was measured. Tumor and liver tissues were fixed in formalin or frozen immediately. Paraffin-embedded tissue sections were dewaxed and hydrated. Liver sections were stained with H&E staining according to the manufacturer’s instructions. The expression of HMGCS1 (1:100; Proteintech, 17643-1-AP), ACSL4 (1:100, Proteintech, 22401-1-AP), cleaved caspase-3 (1:100, ZEN-BIOSCIENCE), Ki67 in the tissue slices of mice tumors were tested by immunohistochemistry.

### 2.16 Statistical Data Analysis

Data were presented as means ± standard deviation (SD) from triplicate experiments performed in a parallel manner, unless otherwise indicated. Comparisons between the two groups were performed by the Student t test. *p* < .05 was considered statistically significant. Statistical significance difference was defined as *p < .05; **p < .01; ***p < .001.

## 3 Results

### 3.1 Itraconazole Inhibits Proliferation and Induces G2/M Cell Cycle Arrest of Human cSCC Cells

**FIGURE 1:**
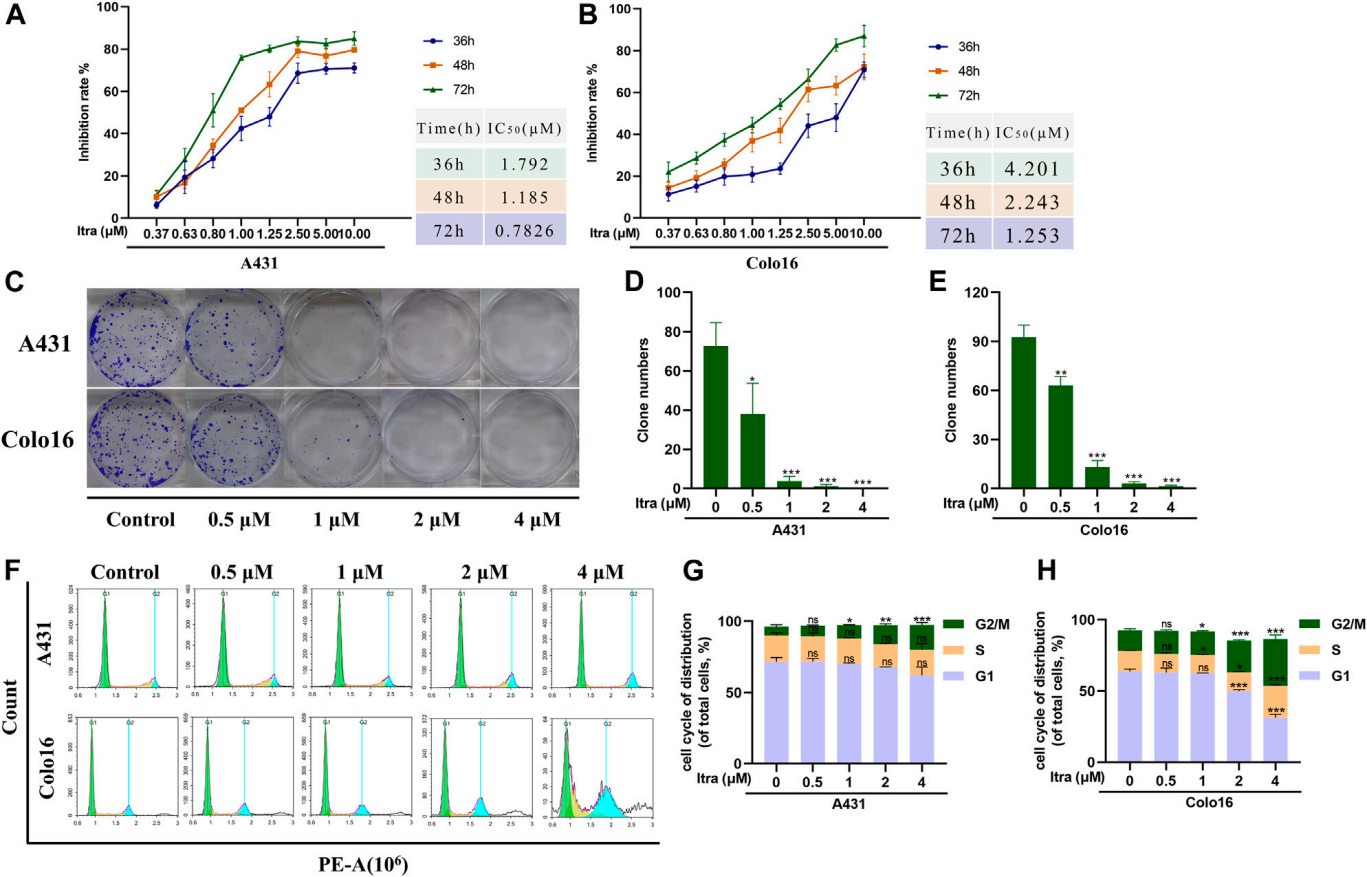
Itraconazole inhibits proliferation and induces G2/M cell cycle arrest of human cSCC cells. **(A,B)** A431 and Colo16 cells were treated with indicated concentrations of itraconazole for 36, 48 and 72 h. Inhibition of cell viability was analyzed by CCK8 assay. **(C–E)** Crystal violet-stained colonies under an inverted microscope. **(F–H)** Cell cycle analysis *via* flow cytometry. [mean ± S.D. (error bars), *n* = 3; *p ≤ .05; **p ≤ .01; ***p ≤ .001, compared with control group].

To explore the effect of itraconazole on the growth of cSCC cells, we used CCK8 assay to detect the proliferation rate of A431 and Colo16 cells after treated with different concentrations of itraconazole. The results of CCK8 assays demonstrated that itraconazole significantly reduces cell viability after incubation for 36 h. Cell viability reductions in A431 (Figure 1A) and Colo16 cells (Figure 1B) were time and concentration-dependent. The colony formation assay confirmed that itraconazole had a dramatical inhibitory effect on A431 and Colo16 cells, and colony numbers in cells with itraconazole were lower than control groups (Figures 1C–E). We used cell cycle analysis to explore the underlying mechanism of proliferation inhibition, and the results showed that itraconazole increased the percentage of cSCC cells in the G2/M phase (Figures 1F–H).

### 3.2 Itraconazole Induces Apoptosis in cSCC Cells

**FIGURE 2:**
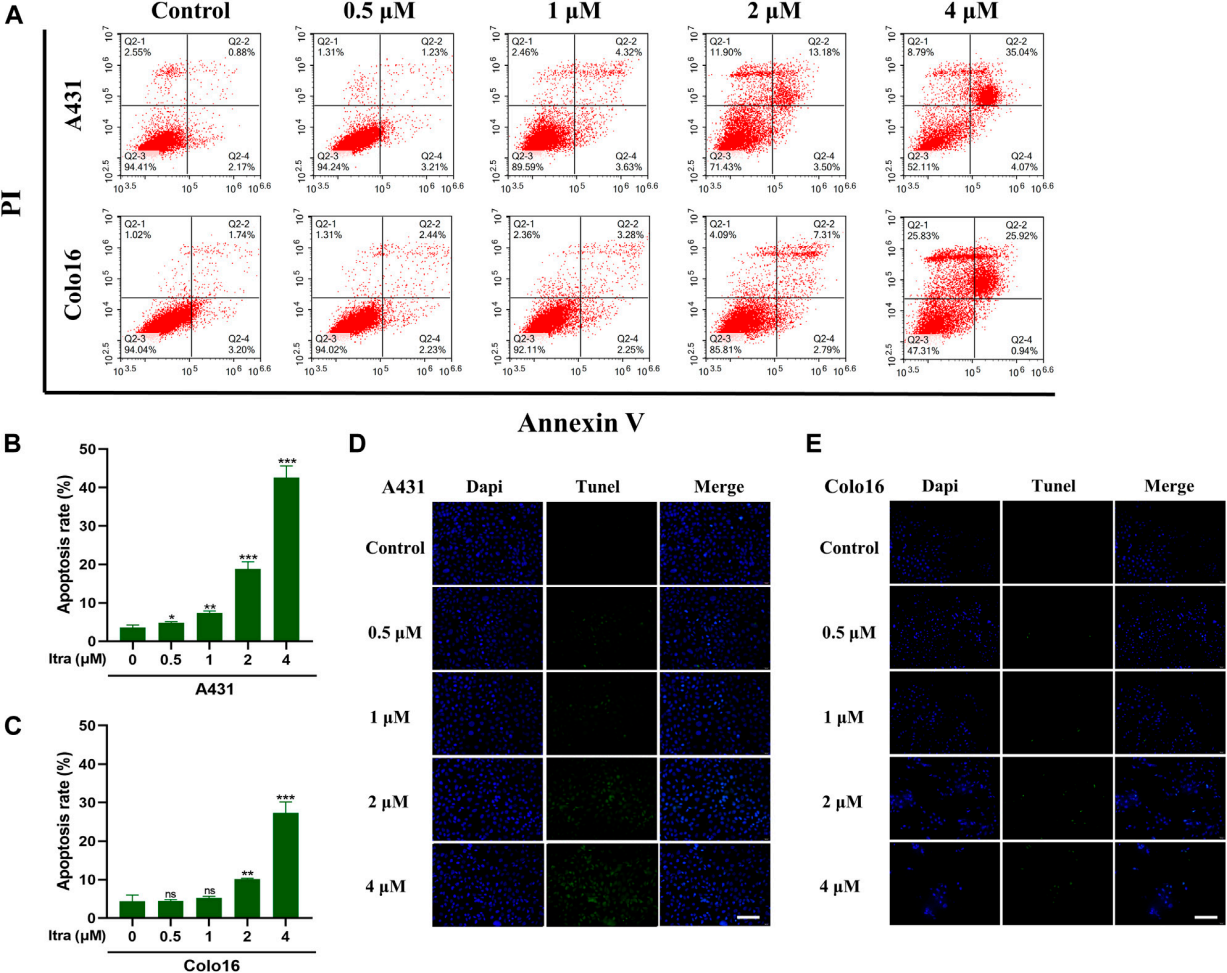
Itraconazole induced apoptosis in cSCC cells. **(A–C)** Cell apoptosis at different itraconazole concentrations, as determined using flow cytometry. [mean ± SD (error bars), *n* = 3; *p ≤ .05; **p ≤ .01; ***p ≤ .001, compared with control group] **(D,E)** TUNEL measurement of apoptosis in A431 and Colo16 cells visualized with a fluorescent microscope (Scale bar: 100 μm).

To investigate whether itraconazole activating apoptosis mechanisms in cSCC cells, we utilized Annexin V/PI assay (Figures 2A–C) and TUNEL staining assay (Figures 2D,E). Results from the assays showed that exposure to itraconazole (48 h) increased the numbers of apoptotic cells in a dose-dependent manner compared with the controls. The results of Annexin V/PI assay showed that with the increase of itraconazole concentration, the total apoptosis rate increased gradually. Apoptotic cells were mainly increased in late apoptotic cells, and there was no significant difference in early apoptotic cells.

### 3.3 Transcriptomic and Proteomic Profiling of A431 Cells Treated With Itraconazole

**FIGURE 3:**
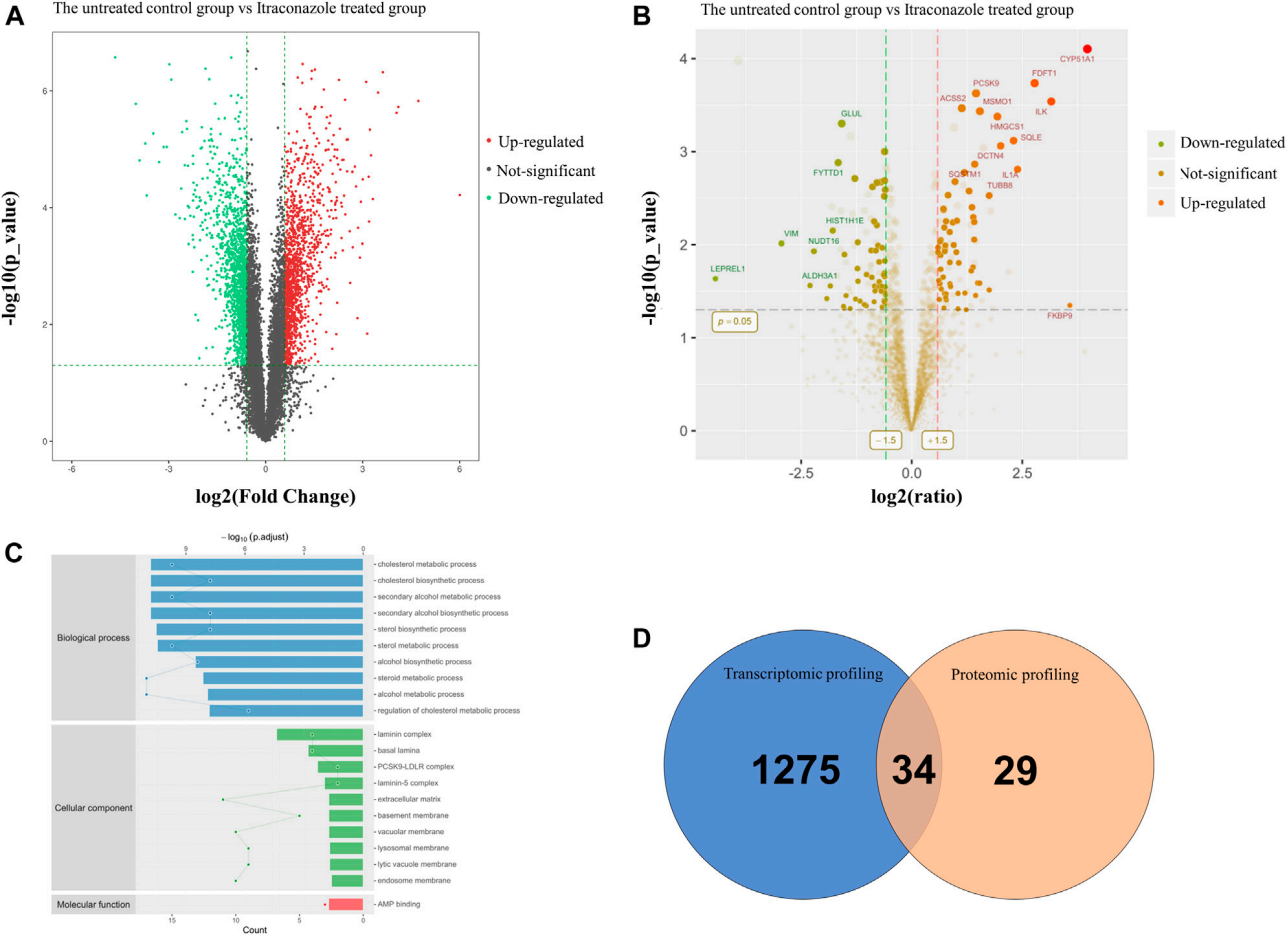
Transcriptomic and proteomic profiling of A431 cells treated with itraconazole. **(A)** Volcano plot of RNA-seq data. The horizontal line represents a *p*-value of .01, whereas the vertical lines correspond to 1.5-fold increase and decrease. **(B)** Volcano plot of iTRAQ data. The horizontal line represents a *p*-value of .05, whereas the vertical lines correspond to 1.5-fold increase and decrease. **(C)** GO enrichment analysis of BP, CC and MF. **(D)** The intersection of transcriptomic and proteomic profiling datasets showed overlap of 34 upregulated proteins in A431 cells.

To understand the transcriptomic alterations in cSCC cells due to itraconazole treatment, we performed RNA-seq analysis of A431 cells when treated with dimethyl sulfoxide (DMSO) (the untreated control group) or with itraconazole (1 μM, 48 h, itraconazole treated group). A total of 1,309 upregulated genes and 1,467 downregulated genes were identified from an RNAseq analysis (Figure 3A). A gene ontology (GO) category enrichment analysis showed that functions including metabolic process were differently enriched in itraconazole treated group and the untreated control group (Supplementary Figures S1A,B). Functional enrichment of transcriptome sequencing data using Kyoto Encyclopedia of Genes and Genomes (KEGG) suggested that the differentially expressed genes were associated with lipid metabolism, NF-kappa B, MAPK, TNF, PI3K/Akt, AMPK and other signaling pathways (Supplementary Figures S1C,D). To further uncover the itraconazole regulatory mechanism in cSCC, we performed proteomic profiling of A431 cells when treated with DMSO (the untreated control group) or with itraconazole (1μM, 48h, itraconazole treated group).63 upregulated proteins and 60 downregulated proteins were identified by iTRAQ analysis (Figure 3B). GO enrichment analysis highlighted over-represented biological processes including cholesterol metabolic process and steroid metabolic process (Figure 3C). The enriched pathways using KEGG included steroid biosynthesis (Supplementary Figure S1E). STRING protein-protein network enrichment analysis of the differentially expressed proteins showed that there are significantly enriched interactions among 23 molecules proteins, including HMGCS1 and ACSL4 (Supplementary Figure S1F). An integrated analysis of transcriptomic and proteomic profiling identified 34 significantly upregulated proteins (Figure 3D), including HMGCS1 and ACLS4. Transcriptomic and proteomic profiling revealed that itraconazole could influence expression of multiple genes and proteins, and diverse several biological pathways in A431 cells.

### 3.4 Itraconazole Affects Human cSCC Development by Regulating HMGCS1 Transcription

**FIGURE 4:**
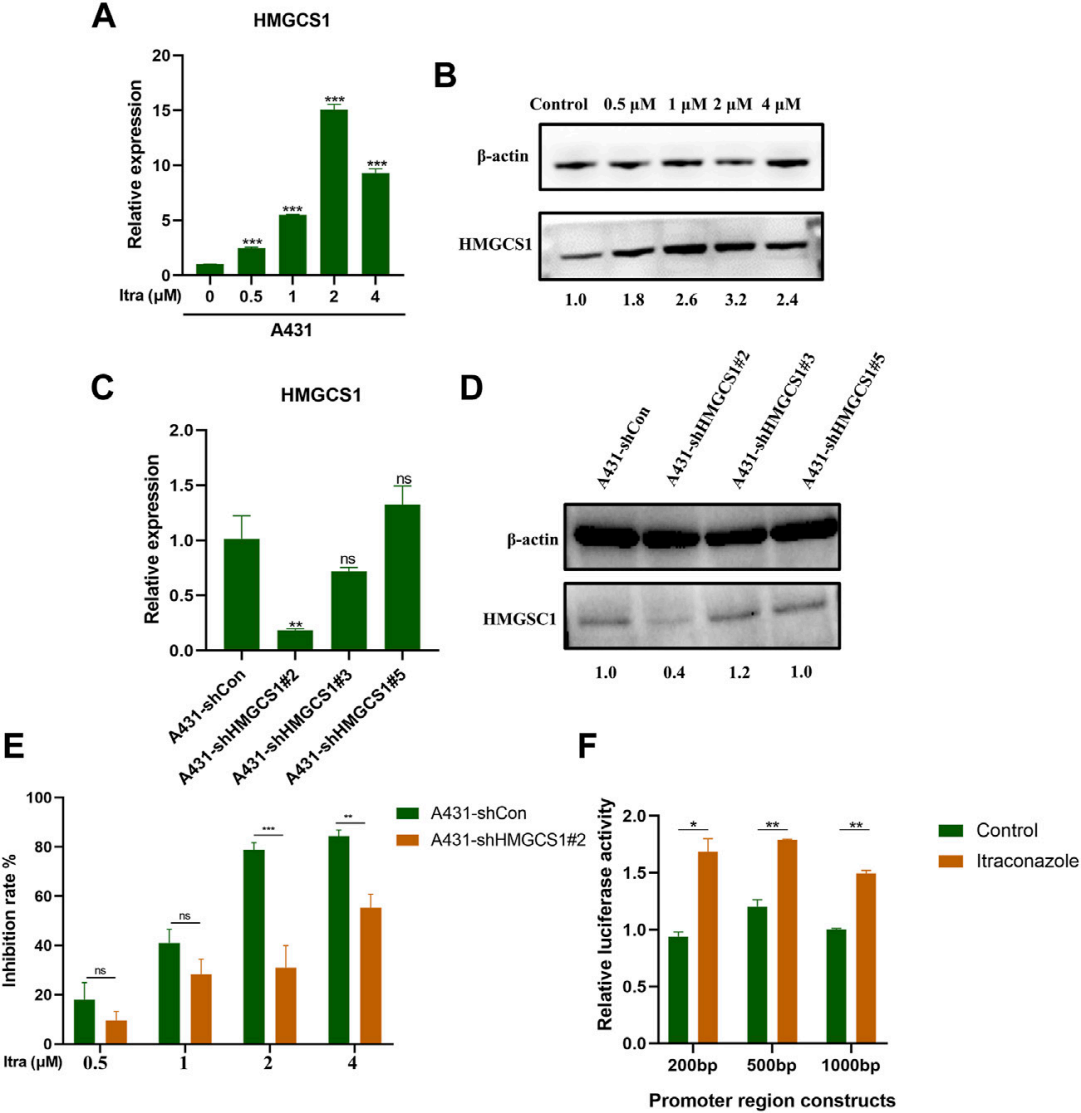
Itraconazole affects human cSCC development by regulating HMGCS1 transcription. **(A)** The relative mRNAs expression levels of HMGCS1 were presented by qRT-PCR. **(B)** The relative proteins expression levels of HMGCS1 were presented by western blot. qRT-PCR analysis **(C)** and western blot **(D)** were performed to detect HMGCS1 expression in A431 cells that were transfected with sh-Con, sh-HMGCS1#2, sh-HMGCS1#3, and sh-HMGCS1#5. **(E)** A431 sh-Con and A431 sh-HMGCS1#2 cells were treated with indicated concentrations of itraconazole for 48 h. Inhibition of cell viability was analyzed by CCK8 assay. **(F)** The relative luciferase activity was detected by dual-luciferase reporter gene activity assay. [mean ± SD (error bars), *n* = 3; *p ≤ .05; **p ≤ .01; ***p ≤ .001, compared with control group].

Based on the transcriptome and proteome results, we were next interested in finding molecular targets of itraconazole in A431 cells. After verification with qRT-PCR (Figure 4A) and western blot (Figure 4B), we confirmed that HMGCS1 was up-regulated in A431 cells treated with itraconazole. We then established HMGCS1 stably knocked-down A431 cell lines. The knockdown efficiency of A431 sh-Con, A431 sh-HMGCS1#2, A431 sh-HMGCS1#3, and A431 sh-HMGCS1#5 was determined by qRT-PCR (Figure 4C) and Western blot (Figure 4D). The highest knockdown efficiency was demonstrated by sh-HMGCS1#2, which was used for the subsequent experiments. CCK8 assay at 48 h showed that knockdown HMGCS1 partly reversed A431 cell proliferation inhibition induced by itraconazole (Figure 4E). 200, 500 or 1,000 bp fragment in the 3′-UTR of HMGCS1 mRNA was respectively inserted downstream of the firefly luciferase gene in a reporter plasmid. As expected, itraconazole dramatically increased the HMGCS1 promoter–luciferase reporter activity compared with the control (Figure 4F).

### 3.5 Itraconazole Induces Ferroptosis Through Upregulating ACSL4 Expression

**FIGURE 5:**
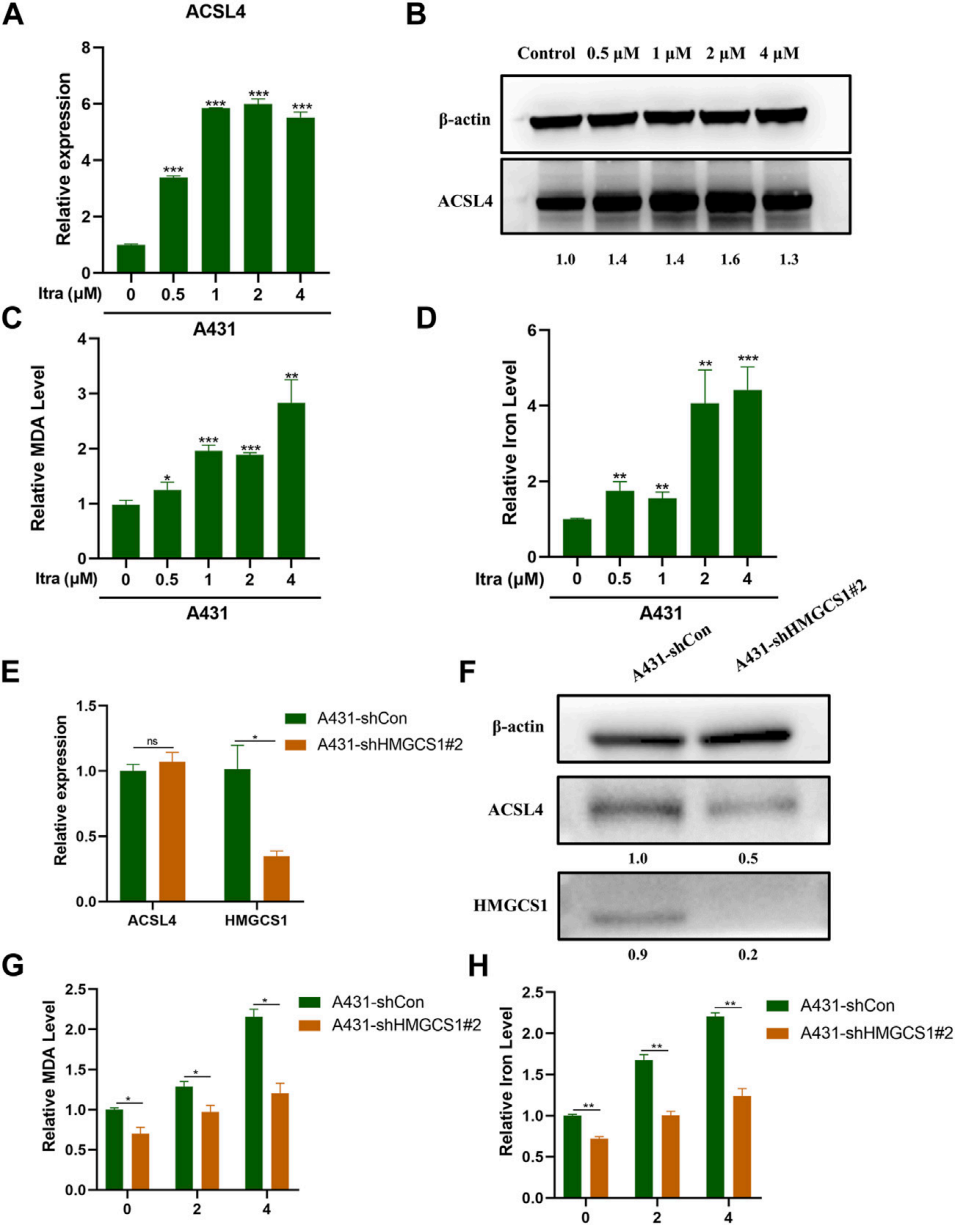
Itraconazole induces ferroptosis through upregulating ACSL4 expression. **(A)** The relative mRNA expression level of ACSL4 was presented by qRT-PCR. **(B)** The protein expression level of ACSL4 was presented by western blot. Relative level of MDA **(C)** and iron **(D)** in A431 cells treated with itraconazole were investigated by specific kits. **(E)** The relative mRNA expression level of ACSL4 and HMGCS1 in A431 sh-Con and A431 sh-HMGCS1#2 was presented by qRT-PCR. **(F)** The protein expression level of ACSL4 and HMGCS1 in A431 sh-Con and A431 sh-HMGCS1#2was presented by western blot. Relative level of MDA **(G)** and iron **(H)** in A431 sh-Con and A431 sh-HMGCS1#2 treated with itraconazole were showed. [mean ± SD (error bars), *n* = 3; *p ≤ .05; **p ≤ .01; ***p ≤ .001, compared with control group].

It was documented that itraconazole could trigger ferroptosis in cancer cells (Xu et al., 2021). ACSL4 is a central contributor and regulator of ferroptosis and it modulates ferroptosis sensitivity (Yuan et al., 2016; Doll et al., 2017). We wished to investigate whether itraconazole could regulate ferroptosis in cSCC cells. Up-regulated ACLS4 in A431 cells treated with itraconazole was confirmed by qRT-PCR analysis (Figure 5A) and western blot (Figure 5B). We analyzed the effects of itraconazole on ROS, MDA and iron accumulation. We determined the regulatory effect of itraconazole (48 h) on ROS production in A431 cells. The results showed that ROS production rates in A431 increased with increasing dose of itraconazole (Supplementary Figures S2A,B). The effects of itraconazole on MDA and iron accumulation were investigated by specific kits. These results displayed that itraconazole led to a marked increase of MDA (Figure 5C) and iron (Figure 5D) in A431 cells. Interestingly, shRNA-mediated silencing of HMGCS1 did not reduce the mRNA expression of ACSL4 in A431 cells (Figure 5E), but reduced the protein expression of ACSL4 in A431 cells (Figure 5F). Silencing of HMGCS1 weakened the promoting effects of itraconazole on MDA (Figure 5G) and iron accumulation (Figure 5H) in A431 cells. Therefore, these findings suggested that itraconazole induced ferroptosis through HMGCS1/ACSL4 axis.

### 3.6 Itraconazole Significantly Inhibits cSCC Cells in a Xenograft Model

**FIGURE 6:**
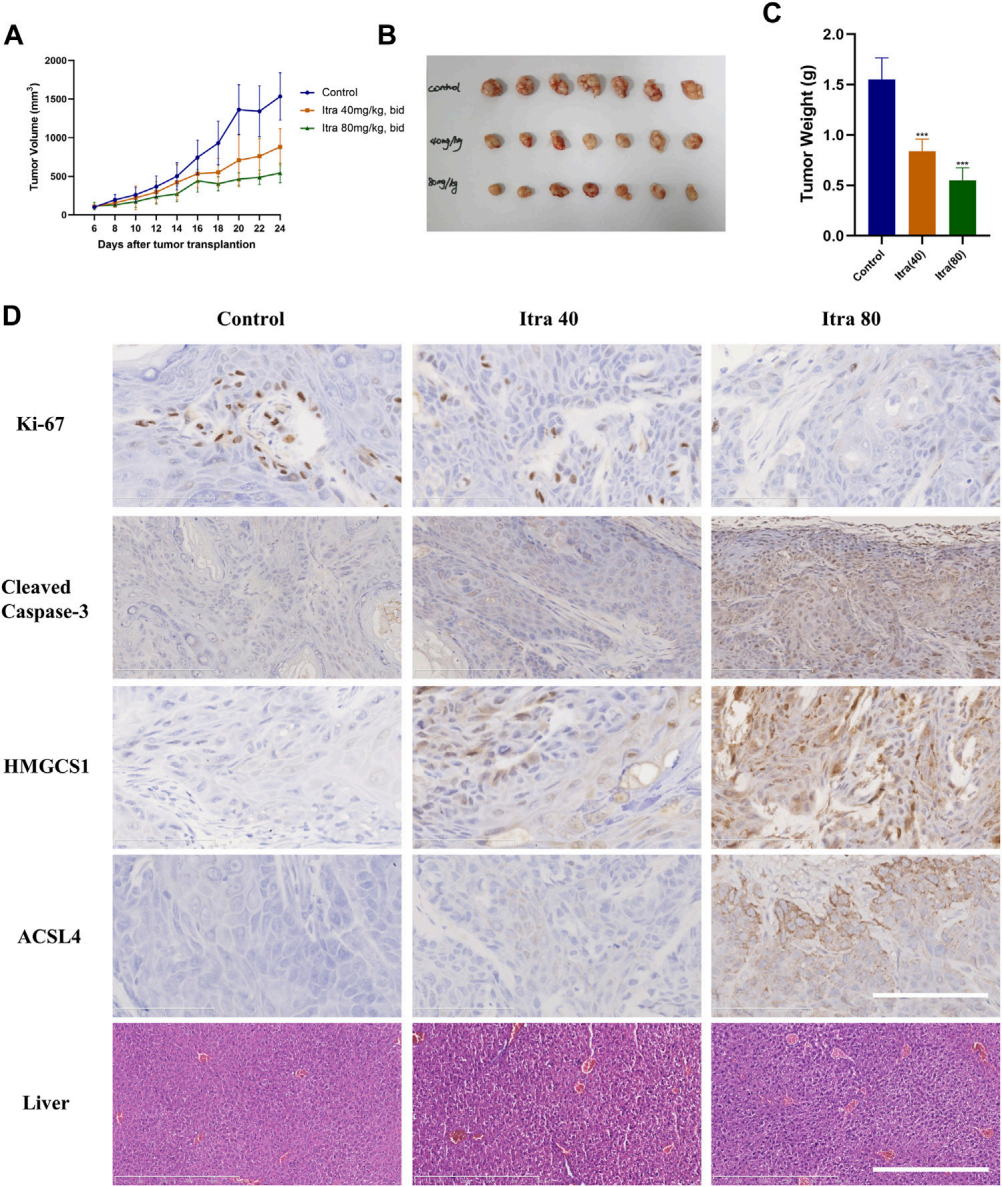
Itraconazole significantly inhibits cSCC cells in a xenograft model. **(A)** Nude mice with A431 subcutaneous tumor xenografts were treated with Normal saline (*n* = 7), 40 mg/kg (*n* = 7) or 80 mg/kg (*n* = 7) itraconazole twice daily by oral gavage for nearly 3 weeks. During drug treatment, tumor volumes were monitored every 2 days. Mice were euthanized at Day-24 after transplantation, tumors were photographed **(B,C)** weighted individually. Representative immunohistochemical analysis of Ki67(D, ×400), cleaved caspase-3 (D, ×400), HMGCS1 (D, ×400), ACSL4 (D, ×400) protein levels of tumor xenografts and H&E staining analysis of liver (D, ×100) in each group (Scale bar of tumor slides: 100 μm; Scale bar of liver slides: 500 μm). *p < .05; **p < .01; ***p < .001; Itra, itraconazole.

To determine the *in vivo* role of itraconazole in cSCC, a xenografted tumor model derived from A431 cells was used. Nude mice with xenografted tumors were randomly divided into three groups, and respectively received treatment with normal saline (control), itraconazole 40 mg/kg twice daily, and itraconazole 80 mg/kg twice daily. Nude mice treated with itraconazole (40 mg/kg, bid; 80 mg/kg, bid) had smaller tumors compared with saline controls (Figures 6A–C), suggesting itraconazole inhibited cSCC cells growths *in vivo*. The expressions of Ki-67 in xenografts were detected by immunohistochemistry to assess cell proliferation. The expressions of Ki-67 were reduced in the low-dose and high-dose itraconazole treatment groups compared with controls, and significantly lower in the high-dose group compared with other groups (Figure 6D). The cleaved caspase-3 staining of the tumor tissues were performed to show if tumor cells were undergoing apoptosis. The results of cleaved caspase-3 staining showed that itraconazole induced cSCC cells apoptosis *in vivo* (Figure 6D). Immunohistochemical staining revealed that the level of HMGCS1 and ACSL4 were significantly dose-dependently increased in itraconazole-treated group (Figure 6D). Liver damage is one of major side effects of itraconazole. To exam the liver necrosis situation, we sliced the liver of the mice and performed H&E staining. There was no significant difference between the livers of the itraconazole-treated group and the control group (Figure 6D). These data demonstrated that itraconazole suppressed cSCC tumor growth *in vivo*. Itraconazole induced cell proliferation and apoptosis, and increased HMGCS1 and ACSL4 expression in xenografted tumor A431 cells.

## 4 Discussion

In this study, we demonstrated that itraconazole inhibited cell proliferation, promoted apoptosis and induced cell cycle arrest in cSCC cells. Functional enrichment analysis suggested that itraconazole could regulate multiple pathways in cSCC cells, including associated with lipid metabolism, NF-κB, PI3K/Akt, AMPK, etc. Itraconazole affects the development of cSCC partly by transcriptional regulating HMGCS1. The protein expression of ACSL4 was reduced after HMGCS1 inhibition. Itraconazole improved the level of ROS, lipid peroxidation products, and iron accumulation. It is possible that HMGCS1/ACSL4 axis was involved in the mechanism of itraconazole inducing ferroptosis.

Some pathways we found in our functional enrichment have previously been demonstrated to be involved in anti-tumor mechanisms of itraconazole in various tumors. Itraconazole inhibited heterodimer formation in NF-κB pathway in the colitis-associated cancer animal models (Kangwan et al., 2016). Itraconazole promoted apoptosis and inhibited proliferation by indirect inhibition of NF-κB pathway (Wei et al., 2020). Itraconazole exerted anti-cancer potential partly through inhibiting PI3K pathway in liver cancer and melanoma (Wenping Wang et al., 2020). Activated AMPK signaling was needed for itraconazole-induced inhibition on esophageal cancer cells (Min-Bin Chen et al., 2018).

HMGCS1 is a regulatory enzyme in the mevalonate pathway. It catalyzes the conversion of acetoacetyl-CoA into 3-hydroxy-3-methylglutaryl-CoA (HMG-CoA), which is the substrate for HMG-CoA reductase (HMGCR) (Kang et al., 2019). Mounting evidence has reported a close relationship between HMGCS1 and cancer initiation and progression (Gruenbacher et al., 2015; Zhao et al., 2017; I-Han Wang et al., 2020; Zhang et al., 2020). A study on cervical squamous cell carcinoma found that HMGCS1 were remarkably downregulated in tumor tissues compared with adjacent tissues, and knockdown of HMGCS1 supported anchorage-independent cancer cell growth. These results suggested that HMGCS1 was a negative regulator in cervical squamous cell carcinoma (Zhang et al., 2020). Where indicated, the mevalonate pathway has been speculated to affect ferroptosis through two mechanisms (Hassannia et al., 2019). Repressing the downstream activity of isopentenyl pyrophosphate (IPP), a direct metabolite of mevalonate, impedes ferroptosis (Hassannia et al., 2019). Conversely, inhibition of HMG-CoA reductase (HMGR) by statins enhances ferroptosis (Shimada et al., 2016). HMGCS1 may involves in mechanisms of mevalonate pathway affecting ferroptosis.

Ferroptosis is driven by lipid peroxidation and regulated by oxidation and antioxidant systems (Dixon et al., 2012). ACSL4 behaves as a crucial regulator in ferroptosis (Yuan et al., 2016). The ACSL4-dependent activation of lipid biosynthesis determined the function of iron-containing enzyme lipoxygenase, which is a major promoter of ferroptosis by producing lipid hydroperoxides (Chen et al., 2020). Not only that, ACSL4 shapes cellular lipid composition as an important node that dictates sensitivity versus resistance to ferroptosis (Doll et al., 2017). Knockdown of ACSL4 makes cells resistant to ferroptosis (Hassannia et al., 2019). In gastric cancer, it acts as a tumor-suppressor gene that inhibits cancer cell proliferation and migration (Ye et al., 2016). ACSL4 facilitates cell sensitivity to chemotherapy in pancreatic cancer (Ye et al., 2020). Transferrin receptor-mediated ROS promotes ferroptosis of human granulosa-like tumor cells via regulating ACSL4 (Lingzhi Zhang et al., 2021). Little is known about the roles of ACSL4 in cSCC. We found that ACSL4 was up-regulated and lipid peroxidation was increased in A431 cells treated with itraconazole. It suggested that itraconazole may suppressed the growth of cSCC through ferroptosis regulated by ACSL4. The protein-protein network enrichment analysis showed HMGCS1 and ACSL4 may have some kind of interaction relationship. HMGCS1 silencing could reduce the protein expression of ACSL4 in A431 cells, without changing the mRNA expression of ACSL4. We tentatively proved that HMGCS1 modulated ACSL4 expression at post-transcriptional levels. Silencing of HMGCS1 weakened the promoting effects of itraconazole on MDA and iron accumulation in cSCC cells. Thus, we inferred that itraconazole may induce ferroptosis via HMGCS1/ACSL4 axis in A431 cells.

The anti-cancer mechanisms of itraconazole might be complicated. This study discovered the effect of itraconazole in cSCC for the first time, and found that its mechanism may include apoptosis and ferroptosis. Certainly, there are some limitations in our work that need to be discussed and further explored. Although we found that the cell proliferation inhibition effect of itraconazole in cSCC was partially reversed by HMGCS1 knockdown, the role of HMGCS1 in cSCC is still not clear. The detailed mechanism underlying the regulation between HMGCS1 and ACSL4 remains to be determined in future works.

In conclusion, our study demonstrated that itraconazole inhibits cSCC significantly *in vitro* and *in vivo*. Our study may bring helpful evidence for repurposing itraconazole in further preclinical and early phase clinical trials for cSCC.

## Data Availability

The original contributions presented in the study are publicly available. This data can be found here: NCBI, GSE191037 and ProteomeXchange, PXD030984.
