# Supplementary material for: Itraconazole Inhibits the Growth of Cutaneous Squamous Cell Carcinoma by Targeting HMGCS1/ACSL4 Axis
Source: Front Pharmacol. 2022 Feb 15;13:828983. doi: 10.3389/fphar.2022.828983 (PMC8886144; doi:10.3389/fphar.2022.828983)
Supplement: Supplementary file 1 [file DataSheet2.docx]

**Supplementary Materials and methods**

**Cell culture and reagents**

Cells were purchased from the China Centre for Type Culture Collection. Cells were cultured in Dulbecco's Modified Eagle Medium (DMEM; Gibco, New York, USA) supplemented with 10% fetal bovine serum (FBS; Gibco, New York, USA) at 37 °C in a cell incubator containing 5% CO2. Cells were grown to 70%-80% confluence in cell culture plates or dishes, and treated under corresponding conditions.

For in vitro usage, itraconazole (S2476, Selleckchem, Houston, TX, USA) was dissolved as a solution in dimethyl sulfoxide (DMSO). For in vivo usage, itraconazole was obtained from Xi'an Janssen Pharmaceutica Ltd. (H20080401).

**Tunel assay**

Briefly, cells for assay were seeded into 6-well plates with climbing slice. The cells were cultured in indicated concentration of itraconazole for 48 h. And then, the cells were washed with PBS, fixed in 4% formaldehyde solution in PBS at 4 °C for 25 min, and permeabilization with diluted proteinase K solution in PBS at room temperature for 5 min. Staining was performed in accordance with the manufacturer protocol. The level of apoptosis was observed under a microscope.

**Real-time PCR analysis**

Total RNA was extracted using RNAiso Plus (Takara Biotechnology Co. Ltd., Shija, Japan). cDNA was synthesized using HiScript II Q RT SuperMix (Vazyme Biotech, Nanjing, China, R223-01). Real-time PCR were performed on a LightCycler® 480 Instrument II (Roche, Basel, Switzerland) using ChamQ SYBR qPCR Master Mix (Vazyme Biotech, Nanjing, China, Q331-02). All experiments were triplicately plated. The primer sequences were:

HMGCS1-F CTCTTGGGATGGACGGTATGC

HMGCS1-R GCTCCAACTCCACCTGTAGG

ACSL4-F TCTGCTTCTGCTGCCCAATT

ACSL4-R CGCCTTCTTGCCAGTCTTTT

GAPDH-F TGACTTCAACAGCGACACCCA

GAPDH-R CACCCTGTTGCTGTAGCCAAA

**Western Blotting Analysis**

Samples were processed as described previously^[29]^. Membranes were blocked with TBST containing 5% BSA for 1 h at room temperature, and then incubated with the following primary antibodies overnight at 4 °C: HMGCS1 (1:1000, Proteintech, 17643-1-AP), ACSL4 (1:1000, Proteintech, 22401-1-AP), β-actin (1:1000, Cell Signaling Technology, 8H10D10). After washing, membranes were incubated for 1 h with appropriate secondary antibodies for 1 h. Proteins were detected using an enhanced chemiluminescence kit (Biorad).

**RNA isolation and transcriptome analyses**

Total RNA was extracted using RNAiso Plus (Takara Biotechnology Co. Ltd., Shija, Japan). RNA was quantified and quality on a NanoDrop ND-1000 instrument. The mRNA was enriched by oligo(dT) magnetic beads. The RNA‐Seq libraries were generated using KAPA Stranded RNA-Seq Library Prep Kit (Illumina) following the manufacturer's instructions. The completed RNA‐Seq libraries were qualified by Agilent 2100 Bioanalyzer (Agilent Technologies, Inc.), and quantified by absolute quantification qPCR method. Sequencing was conducted and raw sequencing data was generated from Illumina NovaSeq 6000. Raw sequencing data that pass the Illumina chastity filter are used for following analysis. Trimmed reads (trimmed 5’, 3’-adaptor bases) were aligned to reference genome. We determined whether the results can be used for subsequent data analysis based on alignment statistical analysis (mapping ratio, rRNA/mtRNA content, and fragment sequence bias). And then, we calculated the expression profiling, differentially expressed genes and differentially expressed transcripts, and predicted the novel genes and transcripts. Differentially expressed mRNAs were identified using the thresholds P<0.05 and fold-change >1.5.

GO analysis (http://www.geneontology.org) was performed to explore the Biological Processes (BP), Molecular Functions (MF), and Cellular Components (CC) of the differentially expressed mRNAs in R or Python environment. KEGG pathway analysis (http://www.genome.jp/kegg/) was performed to analyze whether the differentially expressed mRNAs were enriched in certain biological pathways. The p-values were calculated by Fisher’s exact test to estimate the statistical significance of each GO term and pathway association. P＜0.05 was considered statistically significant and lower P-value implied more significant correlation. The top ten terms of GO and KEGG analysis were all with P<0.05 and false discovery rate (FDR)<0.05.

**Protein extraction and quantification**

Cells were diluted in RIPA buffer containing protease inhibitor cocktail and 1 mM PMSF immediately before use, sonicated to dissolve at 4 °C, and then centrifuged 15 min at 4 °C with top speed. We transferred the supernatant to new tube and keep it on ice. BCA assay, acetone precipitation, re-suspend protein for tryptic digest, cleaning up of SDC, and peptide desalting for base-RP fractionation were performed in order. About 2 µg peptides each sample were separated and then analyzed with a nano-UPLC (EASY-nLC1200) coupled to Q-Exactive mass spectrometry (Thermo Finnigan). The peptides were separated using a reversed-phase column (100 µm, ID × 15 cm, Reprosil-Pur 120 C18-AQ, 1.9 µm, Dr. Math). We performed data dependent acquisition in profile and positive mode with Orbitrap analyzer at a resolution of 70,000 (@200 m/z).

MaxQuant (Version 1.5.6.0) was used to analyze raw MS files. The protein sequence database (Uniprot_organism_2016_09) was downloaded from UNIPROT. This database and its reverse decoy were searched against using MaxQuant software. The quantification type was LFQ with match between run and iBAQ; Trypsin was considered as specific enzyme with up to 3 miss cleavage; Acetyl [protein N-term] and Oxidation [M] were set as variable modification that max number of modifications per peptide is 3, while Carbamidomethyl as fixed modification; FDR of both peptide and protein had to be less than 0.01, and quantification work only used unique & razor peptides. Other parameters were reserved as default. Samples were standardized to make the total protein or median of each group consistent. Differentially expressed protein (DEP) were identified using a fold change > 1.5, and the presence of least 2 unique peptides with a P value <0.05. DEPs were analyzed to two key databases (KEGG and GO) as described previously. The interaction network for the DEPs were created with STRING database (<http://string-db.org/cgi/>).
