# Supplementary material for: Itraconazole Inhibits the Growth of Cutaneous Squamous Cell Carcinoma by Targeting HMGCS1/ACSL4 Axis
Source: Front Pharmacol. 2022 Feb 15;13:828983. doi: 10.3389/fphar.2022.828983 (PMC8886144; doi:10.3389/fphar.2022.828983)
Supplement: Supplementary file 2 [file DataSheet1.docx]

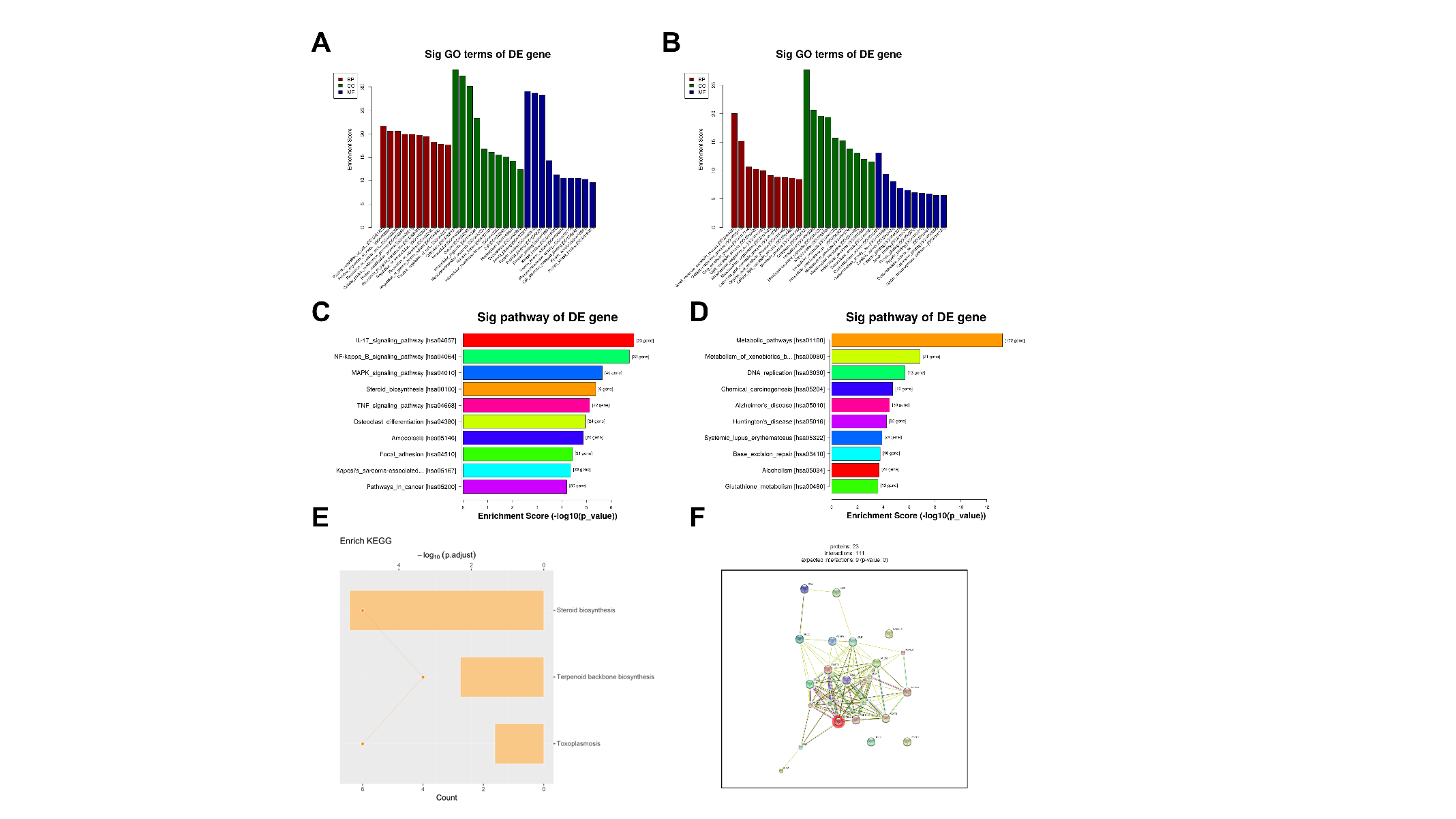


Fig. S1 The top 10 GO terms that associated with up regulated mRNAs (A) and down regulated mRNAs (B) are presented. The top 10 pathways of upregulated mRNAs (C) and downregulated mRNAs (D) differentially expressed mRNAs are presented. DE, differentially expressed; GO, gene ontology; BP, biological process; CC, cellular component; MF, molecular function. (E) The enrichment pathways using KEGG pathway analysis in proteomic profiling are presented. (F) Protein-protein interaction network generated by STRING.


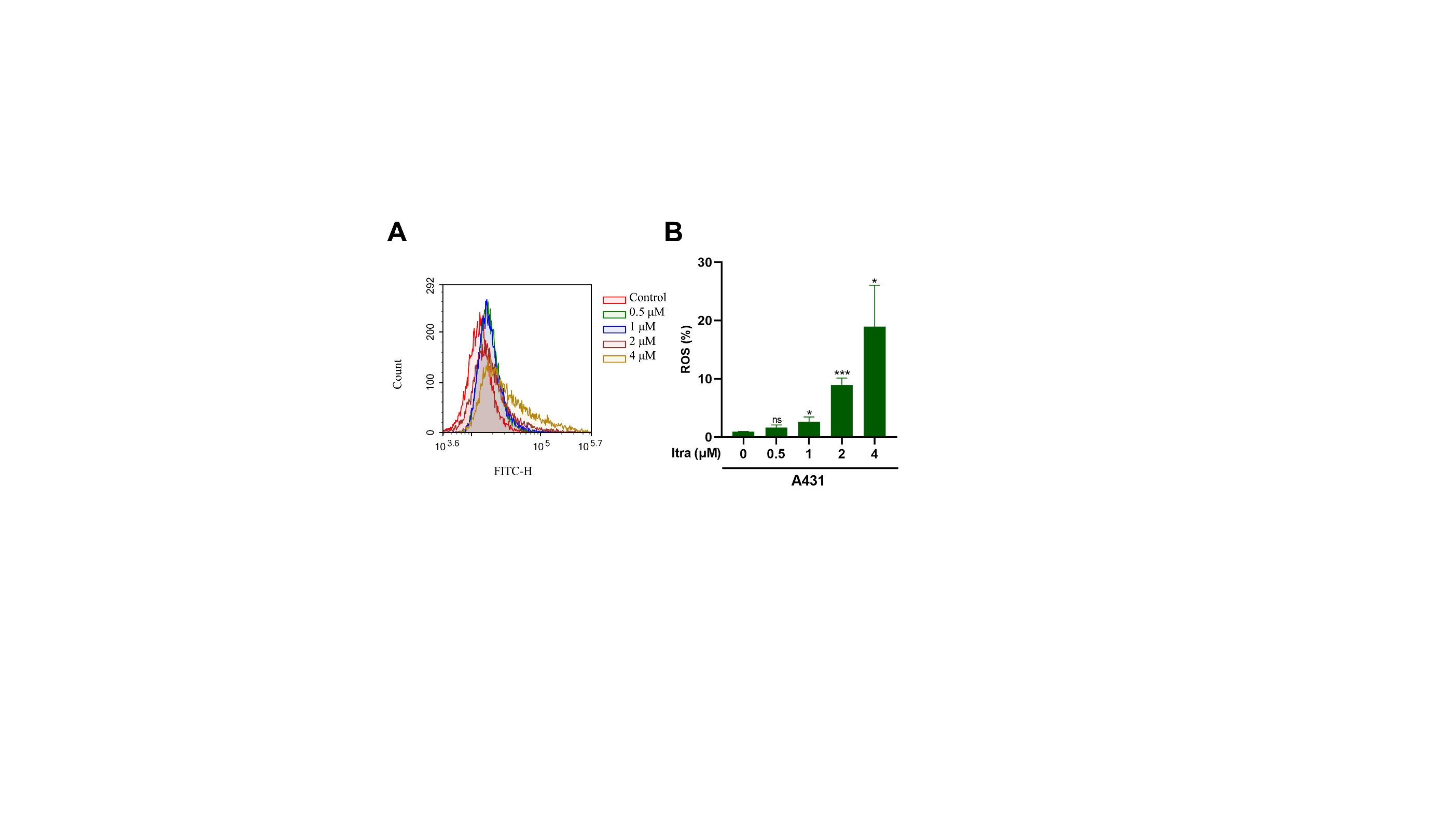


Fig. S2 The ROS production rates of A431 cells treated with itraconazole. Changes in ratio of ROS generation in A431 cells under different concentrations of itraconazole, relative to the normal group.
